# Development and Histopathological Characterization of Tumorgraft Models of Pancreatic Ductal Adenocarcinoma

**DOI:** 10.1371/journal.pone.0078183

**Published:** 2013-10-23

**Authors:** Patrick L. Garcia, Leona N. Council, John D. Christein, J. Pablo Arnoletti, Marty J. Heslin, Tracy L. Gamblin, Joseph H. Richardson, Mary-Ann Bjornsti, Karina J. Yoon

**Affiliations:** 1 Department of Pharmacology and Toxicology, University of Alabama at Birmingham, Birmingham, Alabama, United States of America; 2 Division of Anatomic Pathology, Department of Pathology, University of Alabama at Birmingham, Birmingham, Alabama, United States of America; 3 Department of Medicine, University of Alabama at Birmingham, Birmingham, Alabama, United States of America; 4 Division of General Surgery, Surgical Oncology, Department of Surgery, University of Alabama at Birmingham, Alabama, United States of America; University of South Alabama Mitchell Cancer Institute, United States of America

## Abstract

Pancreatic cancer is the one of the deadliest of all malignancies. The five year survival rate for patients with this disease is 3-5%. Thus, there is a compelling need for novel therapeutic strategies to improve the clinical outcome for patients with pancreatic cancer.  Several groups have demonstrated for other types of solid tumors that early passage human tumor xenograft models can be used to define some genetic and molecular characteristics of specific human tumors. Published studies also suggest that murine tumorgraft models (early passage xenografts derived from direct implantation of primary tumor specimens) may be useful in identifying compounds with efficacy against specific tumor types.  Because pancreatic cancer is a fatal disease and few well-characterized model systems are available for translational research, we developed and characterized a panel of pancreatic tumorgraft models for biological evaluation and therapeutic drug testing.  Of the 41 primary tumor specimens implanted subcutaneously into mice, 35 produced viable tumorgraft models.  We document the fidelity of histological and morphological characteristics and of KRAS mutation status among primary (F0), F1, and F2 tumors for the twenty models that have progressed to the F3 generation.  Importantly, our procedures produced a take rate of 85%, higher than any reported in the literature. Primary tumor specimens that failed to produce tumorgrafts were those that either contained <10% tumor cells or that were obtained from significantly smaller primary tumors. In view of the fidelity of characteristics of primary tumor specimens through at least the F2 generation in mice, we propose that these tumorgraft models represent a useful tool for identifying critical characteristics of pancreatic tumors and for evaluating potential therapies.

## Introduction

Pancreatic cancer (PC) continues to have one of the poorest prognoses among adult solid tumors. Despite comprising only 2% of all cases of cancer in adult patients, PC is the fourth leading cause of cancer related deaths in both men and women in the United States [1,2]. In 2012, an estimated 43,920 patients were diagnosed with pancreatic cancer, and 37,390 patients died from this disease [2]. The most common form of pancreatic cancer is pancreatic ductal adenocarcinoma (PDAC), which represents ~90% of all cases of pancreatic cancer [3]. For patients diagnosed with this devastating disease, surgical resection offers the only potential cure; however, only 10-20% of cases are resectable [4]. Due to the asymptomatic nature of early stage disease and the lack of reliable screening methods, the majority of patients with PC present with advanced stage or metastatic disease at the time of diagnosis. 

The bulk of work focused on improving our understanding of and our ability to diagnose and treat PC has been carried out using established cell lines and xenografts produced from these cell lines [5-7]. These model systems have multiple shortcomings, largely a result of having been cultured *in vitro* for many generations. Established cell lines virtually all accumulate genotypic or phenotypic changes that confer a survival advantage *in vitro*, undergo clonal expansion, and display alterations in characteristics that depend on the interaction of the tumor cells with adjacent stromal tissue *in vivo*. Consequently, the degree to which these cell lines and the xenografts derived therefrom reflect characteristics of the primary tumors from which they were derived is controversial [8-10]. 

Alternatively, genetically engineered mouse (GEM) models offer several advantages over established cell line-based xenograft models [11]. GEM models are established by introducing amino acid substitutions into oncogenes frequently mutated in specific solid tumors. Mutations in KRAS, TP53, p16, or SMAD4, for example, produce a spectrum of premalignant lesions to fully invasive pancreatic adenocarcinomas [12-16]. These models are useful for studying disease progression as well as the relationship of pancreatic tumor development within the tumor microenvironment. A major disadvantage to GEM models in general, however, is that tumors produced in these models are of murine origin; and it is difficult to determine how closely murine tumors recapitulate human tumors. It is also not known how closely these models reflect the known cellular heterogeneity of primary pancreatic tumors, as well as the heterogeneity of tumor-associated stroma seen in human pancreatic tumors. Furthermore, GEM tumors result from a specific mutation introduced into the mouse genome, and human tumors almost certainly result from multiple genetic alterations [17]. Unfortunately, neither cell line-derived nor GEM models of PDAC have reliably predicted the clinical response to new therapeutic agents [18,19]. We propose that the development of well-characterized preclinical models with characteristics documented to be similar to the primary tumors from which they were derived would provide a useful tool for enhancing our ability to understand, diagnose and treat pancreatic cancer. 

Intuitively, heterogeneous specimens of primary human PDAC tumors propagated for a limited number of passages in immunocompromised mice might retain the genotype and phenotype of tumors of origin; and these models could be used to characterize individual PC tumors, identify characteristics common to PC tumors, and evaluate novel chemotherapeutic agents. While this type of patient-derived xenograft was reported in the literature as early as the1960s and 1970s [20,21], the advantages of these models and their use in drug efficacy testing have only recently been recognized and implemented [22-24]. In establishing patient-derived xenograft models, primary tumor specimens are implanted subcutaneously or orthotopically into mice, without an intermediate step of propagation *in vitro*. In this report, we refer to “tumorgrafts” as tumors produced in immunocompromised mice by subcutaneous implantation of heterogeneous human primary tumor specimens, immediately following surgical resection of primary human pancreatic tumors. Other laboratories have established tumorgraft models from breast, pancreatic, renal, head and neck, and hepatocellular tumors, and some of these models have predicted the clinical response of a specific tumor type to several chemotherapeutic agents [25-29]. However, the degree to which established PDAC tumorgraft models reflect the morphological and/or histological characteristics of their tumors of origin has not been published. The goal of our study was to establish and characterize PC tumorgrafts with which to conduct drug sensitivity studies, as has been done for other tumor types. We established multiple models from primary human PDAC tumors and compared morphological, histological, and genetic features of each tumorgraft with those of its tumor of origin. Here we report that our models retain the histopathological and genetic features of the primary tumors from which they were derived over a minimum of two generations, and that histopathological characteristics of primary tumor specimens can be used to predict tumor growth in mice. Our intended use of these models is to identify specific pathways altered in PDAC and/or biomarkers characteristic of this tumor type and to develop more effective therapeutic regimens for this chemorefractory disease. 

## Materials and Methods

### Ethics Statement: Human subjects

This study included human subjects and all procedures were approved by the University of Alabama at Birmingham Institutional Review Board (IRB approved protocol number: X10xxx8006) in accordance with the guiding ethical principles of the IRB-respect for persons, beneficence and justice, as embodied in the Belmont Report.  Written informed consent was obtained from all human participants after discussions of the procedures and potential risks and benefits prior to study participation. Written informed consent was obtained for use of these samples for this specific research purpose only.  No minors/children were included as participants. Tumor tissue received for implantation into mice was deemed in excess of that needed for standard of care. The consent procedure was approved by the Institutional Review Board committee of the University of Alabama at Birmingham. 

### Ethics Statement: Animal protocols

Animal studies were approved by the University of Alabama at Birmingham Institutional Animal Care and Use Committee (IACUC) and were carried out in accordance with Animal Protocol Number (APN): 1x1009186. Four- to six-week old female CB17^-/-^ SCID mice were purchased from Taconic farms (Germantown, NY) and used as hosts for tumorgraft production. All animals were housed in the AAALAC accredited vivarium at UAB Research Support Building under barrier conditions with 12 hour light/dark cycles and *ad libitum* access to food and water. All mice were monitored for tumor growth daily, and tumors were measured twice a week. Mice were euthanized as soon as animals appeared to be in distress or discomfort. Bedding, chow, and cages were autoclaved and cages changed twice a week.

### Tissue procurement

Pancreatic tumor tissue and normal pancreatic tissue were collected from patients who were undergoing surgical resection for pancreatic cancer at the University of Alabama at Birmingham Hospital (Birmingham, AL), and who had given prior consent per IRB-Approved Protocol #10xxx8006 as described in Ethics Statement. Immediately after resection (within an hour), tumor specimens not needed for diagnostic purposes were placed in M-199 medium (Lonza, Walkersville, MD) supplemented with penicillin G/ streptomycin (Pen/Strep, 50 units/mL; Gibco, Grand Island, NY) for transport to the Department of Pathology where tissue samples were grossly evaluated by a board certified pathologist (LNC) with a specialty in gastrointestinal malignancies, to confirm tissue viability and suitability for implantation. 

### Establishment of first generation (F1) tumorgrafts

Tumor tissue (primary tumor, F0 generation) was washed three times in M-199 media containing penicillin/streptomycin (Pen/Strep, 50 units/mL; Gibco, Grand Island, NY) and the entire specimen was dissected into fragments roughly 5 x 5 x 5 mm in size. Mice were anesthetized with 3% isoflurane (Animal Resources Program, Birmingham, AL) and incision sites were disinfected with cholorohexidine. Using surgical scissors, an incision was made 10 mm above the base of the tail, approximately 5 mm in length. A blunt dissection was then made with scissors over the right flank, creating a pocket in which to place the tumor specimen. A single tumor fragment was implanted into right flank of each of three mice. The incision site was treated with 2 drops of Pen/Strep and closure was completed with Vetbond™ (3M, St. Paul, MN). Mice were placed on a mat warmed to 37°C until they had recovered from anesthesia. Mice were monitored daily for discomfort or distress and for tumor growth. The entire procedure, from resection to pathological evaluation to implantation, was performed in less than 60 minutes. F1 generation tumors became palpable 8-12 weeks after implantation. 

### Tumor measurement and monitoring

Tumors were measured with vernier calipers (Fowler/Slyvac, Newton, MA) and tumor sizes were recorded twice weekly, after tumors had reached ~5 mm in diameter. Tumor volumes were calculated by assuming a perfect sphere and the equation v = (π/6)xd^3^, where d represents the mean diameter. 

### Tumor banking and tissue preservation

Tissue collected from normal pancreas or from primary tumor (F0) or subsequent F1 or F2 generation tumor specimens that were not needed for implantation was banked and preserved for future use by three methods. 1) Snap freezing: Tissue was dissected, placed in cryovials and immediately frozen in liquid nitrogen and stored at -80°C. 2) Viable samples: tumor fragments were placed 5 fragments/vial in 1.5 ml of M-199 + 10% DMSO in cold Mr. Frosty (Fisher Scientific, GA) and transferred to -80°C freezer overnight followed by storage in liquid nitrogen. 3) Formalin fixed paraffin embedded (FFPE) tissue: tissue was placed in 10% formalin, embedded in paraffin by standard methods, and thin-sectioned in the Comparative Pathology Lab (CPL) at UAB.

### Establishment of second generation (F2) tumorgrafts

When F1 tumors reached a volume of 1,000-1,500 mm^3^, they were excised, washed in medium + Pen/Strep, cut into multiple fragments (~5 x 5 x 5 mm each). Tumor fragments from one F1 generation mouse were transplanted into each of four naive mice to produce an F2 generation. Donor mice (bearing F1 tumors) were euthanized by CO_2_ and cervical dislocation and submerged in 70% ethanol. Necropsy was performed on donor mice. Tumor tissue not needed for implantation was banked according to the procedures described above. 

### Histological analysis by hematoxylin/eosin (H&E) staining

FFPE sections were de-paraffinized in two changes of xylene, followed by rehydration in two changes of absolute ethanol, and two changes of 95% and 70% ethanol. Tissue was washed briefly in deionized water and stained with Harris hematoxylin (Fischer-Scientific, Suwannee, GA). Slides were then processed in 0.25% acid alcohol, blued in lithium carbonate, and counterstained with eosin solution (Acros Organics-Thermo Fisher Scientific, Fair Lawn, NJ). Tissue was dehydrated in two changes of 95% and absolute ethanol and cleared in xylene. Photomicrographs were taken using an Olympus BH-2 microscope with DP70 camera operating with DPS-BSW v3.1 software (Center Valley, PA).

### Mutational analysis of the KRAS codons 12 and 13

Genomic DNA was extracted from primary tumor (F0) and corresponding tumorgrafts, using a DNA/RNA extraction kit (EpiCentre, Madison, WI). The DNA sequence of codons 12 and 13 in exon 2 of the KRAS gene was determined using standard PCR/direct sequencing methods [30]. The sequenced fragment was a 214-bp PCR product generated with the primers KRAS F: 5’gtgtgacatgttctaatatagtca3’ and KRAS R:5’gaatggtcctgcaccagtaa3’ and 400 ng of genomic DNA [30]. Primers were designed to anneal to human intronic sequences that flank exon 2, to avoid the amplification of murine KRAS sequences. PCR conditions were: initial denaturation for 1 min at 95°C, denaturation for 30 s at 94°C, annealing for 1 min at 58°C, extension for 1 min at 72°C for 30 cycles, and final extension for 7 min at 72°C. After separation in 2% agarose gels, PCR products were extracted using a gel purification kit (Fermentas-Fisher Scientific, Savana, GA). Purified DNA (reaction products) concentration and quality was determined by ND-1000 spectrophotometer using NanoDrop 3.0.1 software (Coleman Technologies, Inc., Wilmington, DE); 280/260 ratios for all DNA examined ranged from 1.80 to 2.00. Reaction products were sequenced by UAB Center for Aids Research (CFAR) DNA Sequencing Core using ABI 3730 sequencer (Life Technologies, Carlsbad, CA). All PCR products were generated and sequenced twice in the forward and reverse directions, in independent experiments. An electropherogram of each sample was provided by CFAR at UAB and visualized using FinchTV (version1.4.0; www.geospiza.com). Of note, some of the electropherogram tracings depicting results for F0 specimens had attenuated, but readily discernible, peaks corresponding to nucleotide substitutions in the sequence of KRAS codon 12. This type of result is expected for DNA obtained from heterogeneous tissue, as demonstrated by Ogino, et al [31]. This concept is discussed in more details in the Discussion. 

### Statistics

Fisher’s exact tests or paired t test were performed using Prism 5.0 (San Diego, CA). A P value less than 0.05 was considered statistically significant. 

## Results

### Establishment of patient tumor-derived tumorgraft models

Between January and October, 2012 a total of 41 pancreatic adenocarcinoma specimens from 41 individual patients were implanted into immunocompromised mice (Table **1**). Specimens were obtained from the primary pancreatic site (N = 39) or from a metastatic site (N = 2; 1 lymph node; 1 ligament of Treitz). Table **1** summarizes clinical data from the 41 patients who participated in this study. Engraftment was deemed successful if the F0 implant reached approximately 1,000-1,500 mm^3^ in size (F1 generation), a size sufficient to perform serial transplantation. Of the 41 implants, 35 were successfully propagated in mice, for an overall take rate of 85% (Table **2**). Of the 35 implants, all remain viable. To date, 34 of the 35 have produced an F2 generation and 20 of the 34 have produced an F3 generation. Table **2** summarizes the current status of these models. The P values in Table **1** refer to the likelihood that a given characteristic was/was not related to successful propagation in mice. These data were generated using Fisher’s exact test to allow analysis of groups of unequal sizes to be compared with nominal variables (in this case, clinical parameters compared with take/no take). These analyses indicated that the size of the primary tumor from which specimens obtained significantly impacted take rate. Primary tumors measuring ≥ 2.5 cm in at least one dimension (greatest dimension) produced viable tumorgrafts more frequently than smaller tumors (96.6% take rate vs 37.5% take rate, respectively [P<0.0001]. (The percent viable tumor cells present in implanted specimens may have also affected take rate, as discussed below.) Our analyses did not identify a correlation between take rate with patient age or gender, tumor differentiation status (histology) or stage, tumor origin (head, body or tail of the pancreas), presence of lymph node metastases, lymphovascular invasion, or perineural invasion (Fisher’s exact test, Prism 5.0 software, San Diego, CA). The apparent lack of correlation between any of these parameters and take rate may reflect a real biological phenomenon or may be due to insufficient numbers of tumors in some categories to accurately predict the factors that significantly affect take rate. 

**Table 1:** Clinical characteristics of patients from whom specimens were acquired and statistical analyses comparing clinical characteristics and take rate.

Variables	Number of patients	Number of F1 tumors produced (take rate)	Impact of clinical characteristics on take rate
**Age**			
≥ 60	33	28 (84.8%)	P=1.0000 (NS)
< 60	8	7 (87.5%)	
**Gender**			
Male	22	20 (90.9%)	P=0.3899 (NS)
Female	19	15 (78.9%)	
**Histology**			
G1	2	1 (50%)	P=0.2874 (NS)^1^
G2	20	18 (90%)	
G3	11	8 (72.7%)	
G2-G3	6	6 (100%)	
Not known	2	2 (100%)	
**Stage**			
≤ II (I and II)	38	32 (84.2%)	P=1.0000 (NS)
> II (III and IV)	2	2 (100%)	
**Tumor Origin**			
Head	31	25 (80.6%)	P=1.0000 (NS)^2^
Tail	4	4 (100%)	
Body	4	4 (100%)	
Metastasis	2	2 (100%)	
**Lymph Node Metastasis**			
Yes	28	24 (85.7%)	P=0.6548 (NS)
No	11	9 (91.8%)	
**Lymphovascular Invasion**			
Yes	26	25 (96.1%)	P=0.1807 (NS)
No	10	8 (80%)	
Perineural Invasion			
Yes	32	29 (90.6%)	P=0.0587 (NS)
No	7	4 (57.1%)	
**Greatest Dimension**			
≥ 2.5 cm	29	28 (96.6%)	P<0.0001 (S***)
< 2.5 cm	8	3 (37.5%)	

^1^ Comparison between G1 and G2+G3

^2^ Comparison between head and tail; head and body; head and metastasis

P values were generated using Fisher’s exact tests, to relate each characteristic (second column) to take rate (fourth column). NS designates no significant difference. S designates significance at P < 0.05.

**Table 2:** Current status of tumorgraft models.

Total specimens implanted (number)	Current tumorgraft status (number)	Tumor take rate
**Primary tumor specimens (41)**	F1 tumorgrafts produced (35)	F0 → F1: 85% (35/41)
**F1 specimens transplanted (34)**	F2 tumorgrafts produced (34)	F1 → F2: 100% (34/34)
**F2 specimens transplanted (20)**	F3 tumorgrafts produced (20)	F2 → F3: 100% (20/20)

Specimens from tumors of 41 patients with resectable pancreatic cancer (pancreatic site [N=39] or metastatic sites [N=2]) were implanted into mice. Take rates (percent) of specimens that have thus far produced F1, F2, and F3 generation tumors are as indicated in the Table.

### Conservation of KRAS mutations in codon 12 of primary tumors and corresponding tumorgrafts

**Figure 1:**
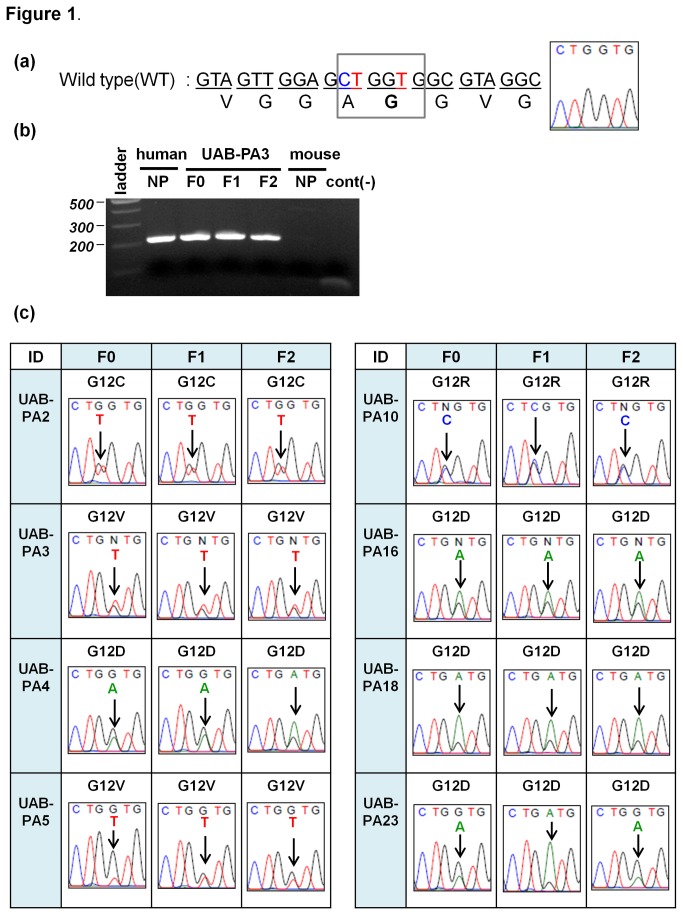
Mutations in codon 12 of the KRAS gene of all twenty primary PDAC tumors (F0) were conserved in the F1 and F2 tumorgrafts derived from each tumor. Electropherograms for eight tumors are shown in this Figure; results for an additional six tumors are shown in Figure S1; results from all twenty F0, F1 and F2 tumors are summarized in Table 3. (**a**) The normal sequence of codon 12 from normal human pancreas DNA (wild type; WT) is GGT (encoding glycine [G]) as shown in the box. (**b**) Representative PCR results using a primer set that anneals to human, but not murine, KRAS sequences. UAB-PA3: NP (normal pancreas), F0 (primary tumor), and F1 and F2 tumorgrafts show readily detectable bands (214 base pairs). Mouse NP (normal pancreas) and negative control (cont [-]) lanes showed no bands. Experimental details are in Methods section. (**c**) Electropherograms show mutations in codon 12 of the KRAS gene in eight primary PDAC tumors (F0) and in the F1 and F2 tumorgrafts derived from each tumor.

KRAS mutation is one of the most common oncogenic mutations in human cancers, including PC [32,33]. Mutations of the KRAS oncogene constitutively activate the KRAS signaling pathway. KRAS mutations are present in approximately 90% of PDAC tumors, with mutations occur most commonly in codon 12 [34]. To evaluate whether key genetic characteristics of F0 specimens were also present in tumografts derived from these specimens, we analyzed twenty tumorgraft models that have progressed to the F3 generation. Based on the availability of sufficient material from F0 tumors and corresponding F1 and F2 tumorgraft specimens, we used polymerase chain reaction (PCR) with human-specific primers (Figures **1a and 1b**) and sequencing techniques to identify the mutational status of codons 12 and 13 in the KRAS gene (Figure **1** and data not shown). No mutations were detected in codon 13 in any of the F0, F1, or F2 specimens analyzed. In contrast, as shown in Table **3**, mutations were present in primary tumor tissue (F0) in codon 12 of the KRAS gene of all specimens analyzed and these mutations were conserved in 100% of F1 and F2 generation tumorgrafts. More specifically, in pancreatic cancer, the most commonly reported KRAS mutation is the substitution of valine (V) or aspartic acid (D) for glycine (G) at position 2 in codon 12 (GGT [encoding G] → GTT [encoding V] or GAT [encoding D]) [35]. Consistent with the literature, 16 of the 20 tumors analyzed contained V (6 cases) or D (10 cases) substitutions at this position (Table **3**, Figures **1c** and **S1**). The other four models analyzed harbored more rare glycine (G) → cysteine (C) or glycine (G) → arginine (R) substitutions at codon 12. Specific KRAS mutations in each of the 20 models evaluated were conserved among F0, F1 and F2 specimens. To then evaluate conservation of KRAS status among tumors of a given generation, we analyzed the sequence of KRAS codons 12 and 13 in tumors from multiple mice bearing F1 and F2 tumors that originated from each of three independent F0 specimens.

**Table 3:** Mutations in codon 12 of the KRAS gene were conserved from primary tumor (F0) through the F2 generation of all twenty tumorgraft models evaluated.

Specimen ID	KRAS codon 12 sequence change (F0: primary tumor)	Mutation conserved in F1 and F2 tumorgrafts	Amino acid substitution
UAB-PA2	GGT → TGT	Yes	G12C
UAB-PA3	GGT → GTT	Yes	G12V
UAB-PA4	GGT → GAT	Yes	G12D
UAB-PA5	GGT → GTT	Yes	G12V
UAB-PA8	GGT → GTT	Yes	G12V
UAB-PA10	GGT → CGT	Yes	G12R
UAB-PA13	GGT → GAT	Yes	G12D
UAB-PA16	GGT → GAT	Yes	G12D
UAB-PA18	GGT → GAT	Yes	G12D
UAB-PA20	GGT → CGT	Yes	G12R
UAB-PA22	GGT → GTT	Yes	G12V
UAB-PA23	GGT → GAT	Yes	G12D
UAB-PA26	GGT → GAT	Yes	G12D
UAB-PA28	GGT → CGT	Yes	G12R
UAB-PA29	GGT → GTT	Yes	G12V
UAB-PA30	GGT → GTT	Yes	G12V
UAB-PA33	GGT → GAT	Yes	G12D
UAB-PA34	GGT → GAT	Yes	G12D
UAB-PA36	GGT → GTT	Yes	G12V
UAB-PA37	GGT → GTT	Yes	G12V

The sequence of codon 12 of KRAS in normal tissue is GGT, encoding glycine (G). Mutations (column 2) and resulting amino acid substitutions (column 4) are indicated in the Table.

### Sister tumorgrafts originating from the same F0 or F1 tumor retained KRAS codon 12 and 13 mutational status

**Figure 2:**
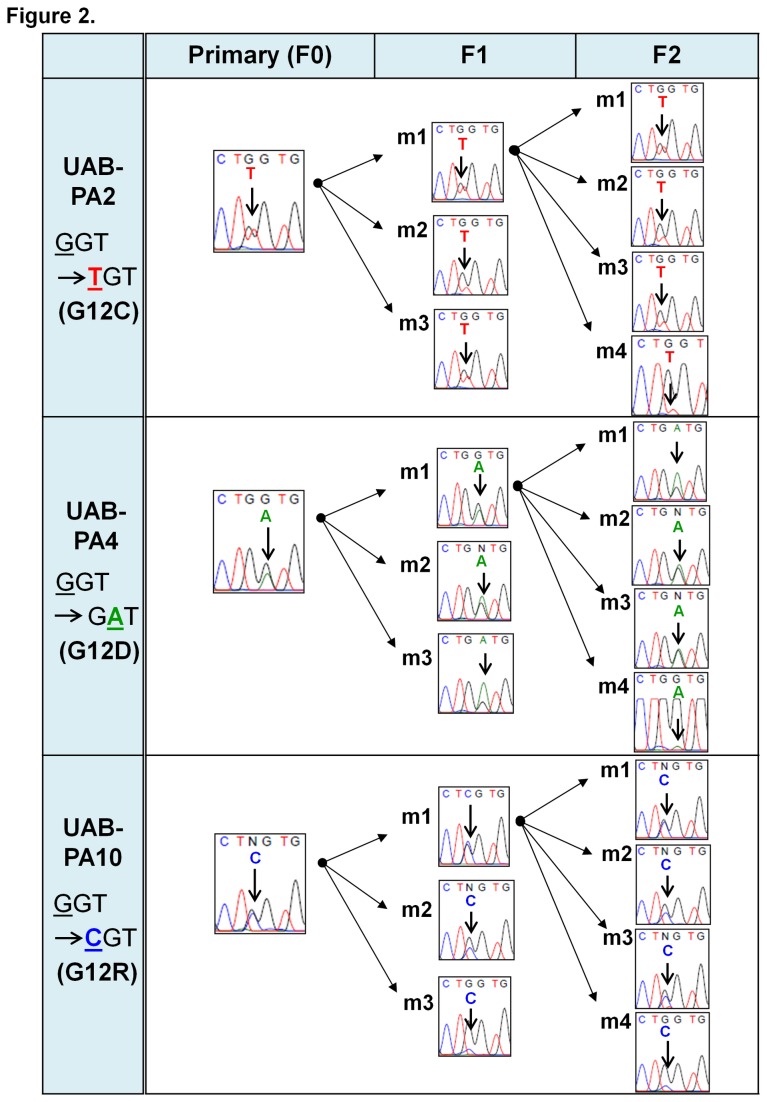
Sister tumorgrafts originating from the same F0 or F1 tumor retained KRAS codon 12 mutational status. Electropherograms demonstrate that sister tumorgrafts (from mouse 1 [m1], mouse 2 [m2], mouse 3 [m3], and mouse 4 [m4] of each set) from all three models (UAB-PA2; UAB-PA4; UAB-PA10) conserved KRAS codon 12 status in F0, F1 and F2 tumors. Experimental details are reported in Materials and Methods.

We randomly selected three independent primary (F0) tumors, and analyzed the sequence of KRAS codons 12 and 13 of these tumors. We also analyzed three F1 progeny derived from each F0 tumor (three “sets” of three F1 sister tumorgrafts) and four F2 progeny of one tumor from each set of F1 tumors (three “sets” of four F4 sister tumorgrafts) (Figure **2**). The data demonstrate that sister tumorgrafts (from mouse 1 [m1], mouse 2 [m2], mouse 3 [m3], and mouse 4 [m4] of each set) from all three models (UAB-PA2; UAB-PA4; UAB-PA10) conserved KRAS codon 12 status. As in previous analyses, no mutations in codon 13 were identified (data not shown). These results indicate that tumorgrafts in this study having the same origin displayed fidelity of KRAS status within tumors of a given generation.

### F1 and F2 generation tumorgraft tissues are morphologically similar to F0 primary tumor tissue of origin

Having demonstrated fidelity in F0, F1, and F2 tissue specimens with respect to the mutation status of codon 12 of KRAS, we next addressed fidelity of tissue morphology of the same twenty models. As shown in Table **1**, a majority of these models were derived from stage IIA or IIB tumors, stages of PDAC regarded as resectable disease. 

Histology was evaluated using criteria for clinical staging and diagnosis of PDAC, a subtype that accounts for 90% of all cases of PC. PDAC displays characteristic lesions that have at least one of three specific histological traits: pancreatic intraepithelial neoplasia (PanIN), intraductal papillary mucinous neoplasm (IPMN), or mucinous cystic neoplasia (MCN) [36]. Among these, the most common and well-characterized precursor lesion is PanIN. PanIN lesions, in turn, are subdivided into four grades (PanIN-1A, PanIN-1B, PanIN-2, and PanIN-3) based on degree of dysplasia, as reflected by cytologic atypia and architectural changes [37]. Briefly, PanIN-1A and 1B are characterized by tall columnar cells and mucin production. PanIN-1B lesions have a papillary (or micropapillary) architecture. PanIN-2 lesions produce mucin, and have predominant nuclear abnormalities. Histological abnormalities of PanIN-2 type lesions include cytonuclear atypia, nuclear crowding, and nuclear abnormalities (most commonly nuclear enlargement). PanIN-3 lesions are characterized by many of the cytologic hallmarks of solid tumors in general, such as cribriforming, loss of nuclear polarity, nuclear atypia, luminal necrosis, abnormal mitoses, and budding off of groups in small clusters into the ductal lumen. Eventually, progressive development of dysplasia by all four subclasses of PanIN precursor lesions produces invasive pancreatic adenocarcinoma. 

**Figure 3:**
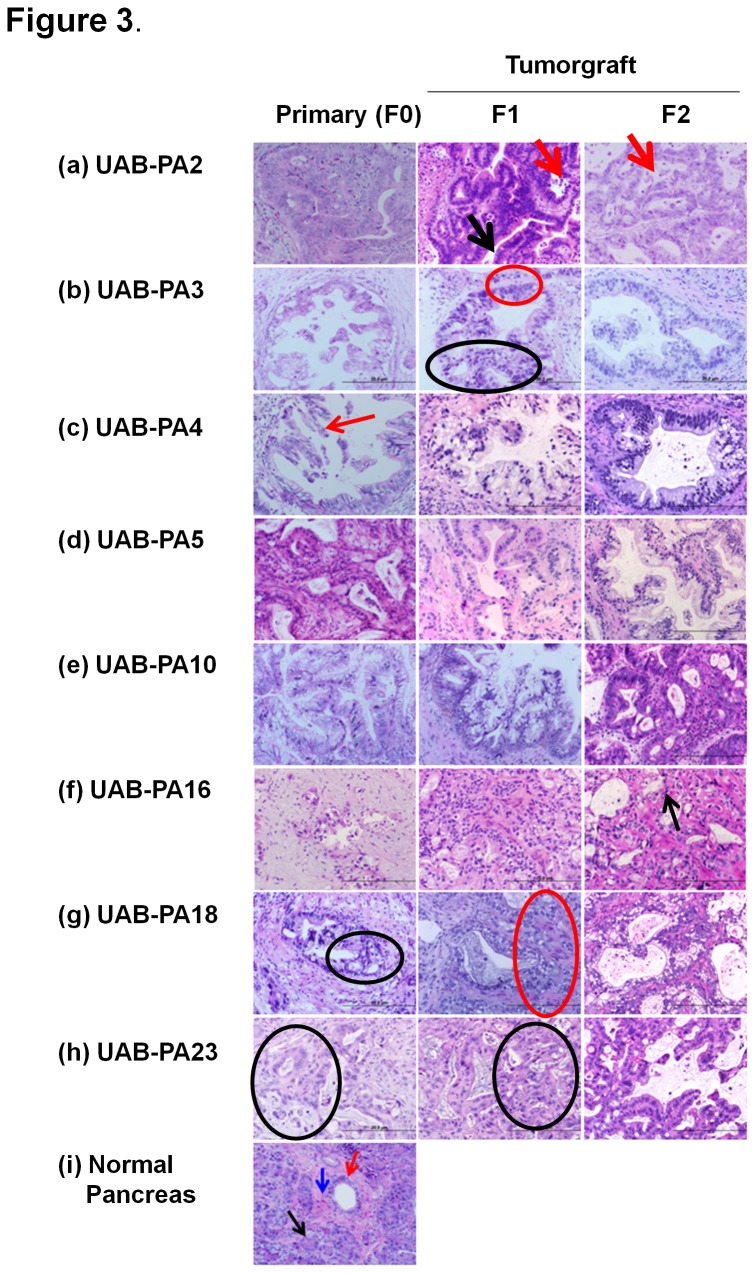
Histological evaluation of eight F1 and F2 tumorgrafts demonstrates morphological fidelity of these tumors with the F0 tumors from which they were derived. Detailed descriptions of histological characteristics are included in the Results section. Histological evaluation of an additional twelve models are presented and described in the Supporting Information; Figure S2.

Summaries of the histological and morphological characteristics (by LNC) of each of the twenty tumorgraft models that have progressed to the F3 passage are based on: degree of differentiation, atypical gland formation, cytonuclear atypia, and nuclear abnormalities (e.g., nuclear:cytoplasmic ratio). These characteristics comprise the predominant indicators of PDAC malignancy in primary patient tumors (F0) and are used to describe the degree to which tumorgraft (F1, F2) progeny reflected characteristics of F0 tissue. We defined the degree of differentiation based on the presence or absence of glandular formation. Tumors containing gland tissue were designated as well-differentiated or moderately differentiated. Tissue closely resembling normal pancreatic (NP) tissue is routinely designated as well-differentiated. However, none of our tumor specimens (presented in Figures **3** and **S2**) fulfilled this criterion; all specimens contained moderately to poorly differentiated tumor cells. Tumors with complex histology, for example with cystic, cribriformed, solid nest, or malignant cytology were designated as moderately differentiated. Tumors that had not formed glands, as evidenced by sheets of cells, sarcomatoids differentiation, or anaplastic cells, were designated as poorly differentiated. In general, all tumorgraft models evaluated retained the essential characteristics of primary pancreatic adenocarcinomas from the F0 through the F2 generation. H&E images and detailed histological characterization of eight tumors are shown below (Figure **3**, panels **a-h**); similar analyses of the remaining twelve tumors are depicted and described in Supporting Information (Figure **S2**, panels **j**-**u**). 

#### Tumor UAB-PA2 (a)

F0, F1 and F2 generation tissues are all moderately differentiated and similar in terms of intact gland formation. F0 and F1 tissues are remarkably similar with respect to glandular morphology and cytology. The F2 tissue displays more cytologic atypia, specifically an increased nuclear:cytoplasmic (N:C) ratio and also more nuclear pleomorphism. F1 and F2 tissues show more apparent necrotic debris (red arrow) within the glandular lumina (black arrow) than the F0 tissue from which they were derived. F0 through F2 specimens all show characteristics typical of invasive PDAC with PanIN-3 features. 

#### Tumor UAB-PA3 (b)

Tissues from all three types of specimens (F0, F1, and F2) are moderately differentiated, with comparable amounts of peritumoral stroma. There is marked increase in cytologic atypia (particularly increased N:C ratio) in F1 compared to F0 tissues. There is also a noticeable increase in architectural complexity, with F1 tissue glands showing more crowding (red circle) and increased cribriforming (black circle). All three generations display similar cytology and glandular morphology. F1 and F2 tissues are most similar to each other in terms of cytology. Also, compared to F0, F1 and F2 tissues of UAB-PA3 display more homogeneous nuclear features (hyperchromasia and polarity) and more homogeneous N:C ratios. F0 through F2 display PDAC with PanIN-3 characteristics. 

#### Tumor UAB-PA4 (c)

Peritumoral (or periglandular) stroma and moderate differentiation is maintained from F0 through F2 tissues; however, F0 tissue has more apparent architectural and cytologic atypia than F1 tissue. The F1 tissue acquired clear cell features and increased nuclear polarity but still maintains the micropapillations (red arrow) seen in F0 tissue. As was observed for Tumor UAB-PA3, F1 and F2 tissues of Tumor UAB-PA4 are more similar in morphology to each other than to the F0 specimen, in that they have clear cell-like features and nuclear features. F2 tissue has decreased N:C ratio compared to F1 tissue and also has fewer micropapillations. While some unique features distinguish the specimens, F0 through F2 specimens are all classified as PDAC with PanIN-3 characteristics. 

#### Tumor UAB-PA5 (d)

F0 through F2 tissues are all moderately differentiated with intact but complex gland formation. All have a moderate amount of peritumoral stroma. Cytologically, all three generations show apical cytoplasmic clearing and conserved nuclear polarity. The N:C ratio is higher in F0 and F1 tissues than in F2 tissue; but all three types of specimens represent PDAC tumors with PanIN-2 features. 

#### Tumor UAB-PA10 (e)

F0 through F2 tissues display moderate differentiation. The F2 tissue has less apparent peritumoral stroma, compared to F0 and F1 tissues. There is overall maintenance of architectural integrity between F0 and F1 tissues. Both have complex gland formation with cribriforming. Compared to F0 and F1, F2 tissues has an increased N:C ratio and loss of nuclear polarity. Although overall degree of differentiation is maintained in terms of gland formation/architecture, the cytology of F2 tissue is higher grade than F0 and F1 tissues. F0 through F2 are classified as PDAC with PanIN-3 features. 

#### Tumor UAB-PA16 (f)

F1 and F2 tissues are similar in terms of cellularity and cytology with F2 tissue showing slightly more cytologic atypia (increased loss of nuclear polarity) (black arrow indicates cell with this characteristic). There is progressive loss of peritumoral stroma from F0 to F2 tissues, but all are considered moderately differentiated PDAC. 

#### Tumor UAB-PA18 (g)

F0 through F2 tissues show moderate differentiation with slightly greater peritumoral stroma demonstrated in F0 and F2 tissues than in F1 tissue. In terms of glandular morphology, F0 and F1 tissues are very similar. Both show a wide array of architectural atypia including cribriforming (black circle). The F1 specimen shows more glands/glandular crowding (red circle) and fewer small glands. F1 and F2 tissues show slightly increased cytologic atypia as compared to F0 tissue. F0 through F2 are classified as PDAC with PanIN-3 features. 

#### Tumor UAB-PA23 (h)

F0 through F2 tissues show moderate differentiation. There is progressively less peritumoral stroma in F1 tumors compared to F0 and in F2 tumors compared to F1. F0 and F1 tissues are very similar in architecture and cytology. Both show complex glandular architecture (black circle) and increased nuclear pleomorphism. F1 tissue has increased glandular crowding, and F0 tissue has a more fibrotic/desmoplastic background, compared to F1 and F2 tissue. F0 through F2 tissues show similar architectural and cytologic morphology. F2 tissue contained less mucin and necrotic debris than F0 and F1 tissue. F0, F1 and F2 specimens are all classified as PDAC with PanIN-3 features. 

#### Normal pancreas (i)

The photomicrograph in the Figure **3** shows normal pancreatic parenchyma with intact acinar tissue (black arrow) with a moderate amount of fibrous stroma (blue arrow) and a central pancreatic duct (red arrow).

In summary, morphological and histological characteristics and degree of differentiation were well preserved among F0, F1 and F2 tissue for each of the twenty models evaluated. Minor variations among the generations of a given model were noted, but all tumorgraft models (F1 and F2 generations) shown in Figures **3** and **S2** retained the most essential characteristics of pancreatic adenocarcinomas. 

### The F3 generation of UAB-PA2 tumorgrafts retains the histology of the F0 generation

**Figure 4:**
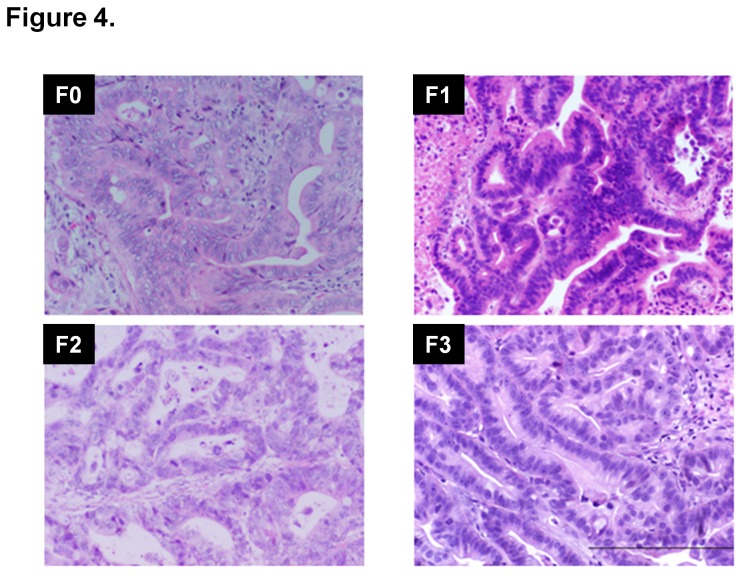
Three generations of tumorgraft UAB-PA2 retain morphology similar to that of the primary tumor. UAB-PA2 tumor was serially passaged to the third generation (F3), and the morphology of H&E stained sections of tumors compared. The Results section contains details of histological analyses.

To date, we have sufficient material for histologic analysis from the F3 generation of only model UAB-PA2. Notably, as shown in Figure **4**, the F3 tissues have characteristics very similar to those of F0 UAB-PA2 tissue. All generations, F0, F1, F2 and F3 tissues show equivalent N:C ratios, display consistent cellular differentiation, and have similar histological features. This *in vivo* F3 generation preserved the histological features of its tumor of origin. We next compared the growth patterns of F1 and F2 generations of each of these twenty models. 

### Comparison of the growth patterns of F1 with F2 generation tumorgrafts

**Figure 5:**
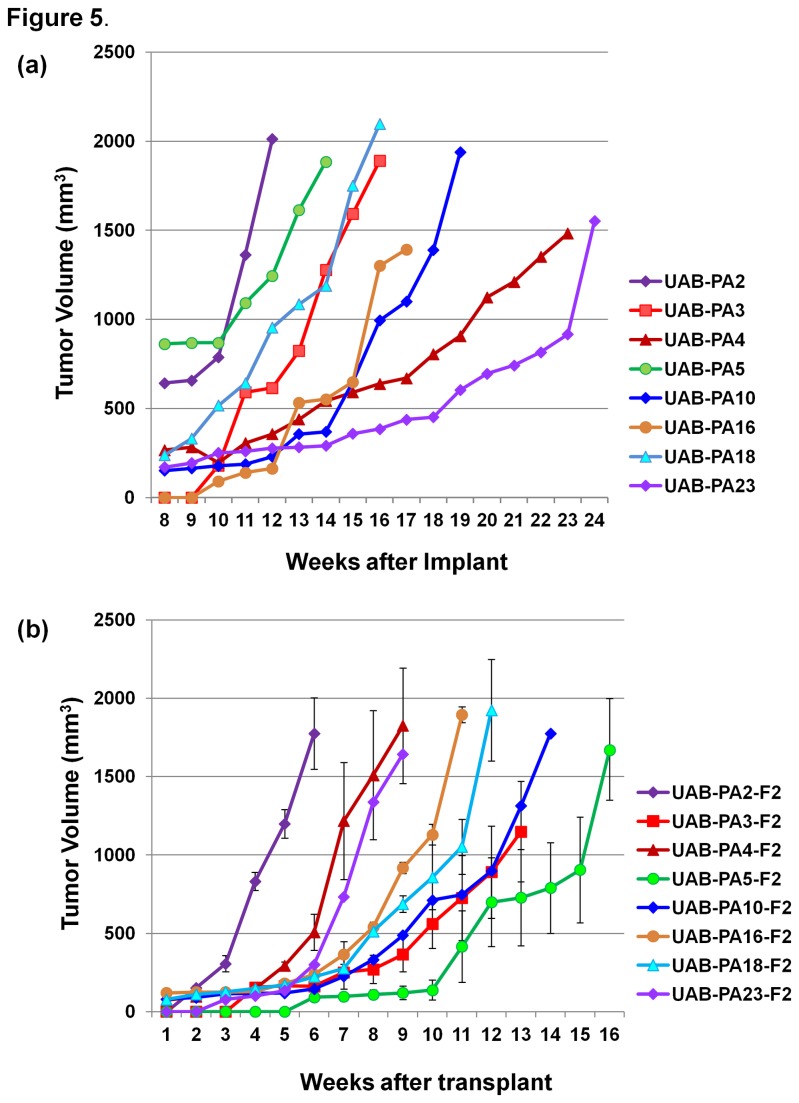
Tumorgraft growth curves. (**a**) First generation (F1) tumorgraft growth curves. (**b**) Second generation (F2) tumorgraft growth curves. Tumors were measured using vernier calipers once a week until tumor volumes reached ~1,500 mm^3^. Details of the procedure and calculations to determine tumor volume are as described in Methods. The graph in the figure was generated using Microsoft excel software.

The growth curves of F1 tumors produced by individual F0 tumor specimens following implantation into mice are shown in Figures **5a** **, S3a and S3c**. The F1 tumors demonstrated a range of growth rates that differed by ~12-fold, based on an approximated slope during exponential growth (range of slope values: 28 [UAB-PA8] to 344 [UAB-PA2]). F1 tumors reached ~1,500 mm^3^ 11-38 weeks after implantation. 

F2 tumor growth curves (Figures **5b** **, S3b and S3d**), generated from data obtained from 2-4 mice per tumor model, show that while F2 tumors grew at rates similar to each other, differences were observed in the interval between implantation and measurable tumor progression (lag phase). Compare, for example, the slopes of the lines representing UAB-PA2-F2 tumor growth compared to UAB-PA16-F2 after a ~7-week lag period. F2 tumors reached ~1,500 mm^3^ 6-25 weeks after transplantation. 

### Cryopreserved tumorgraft transplant shows the growth similar to that following direct transplantation of non-frozen specimen

**Figure 6:**
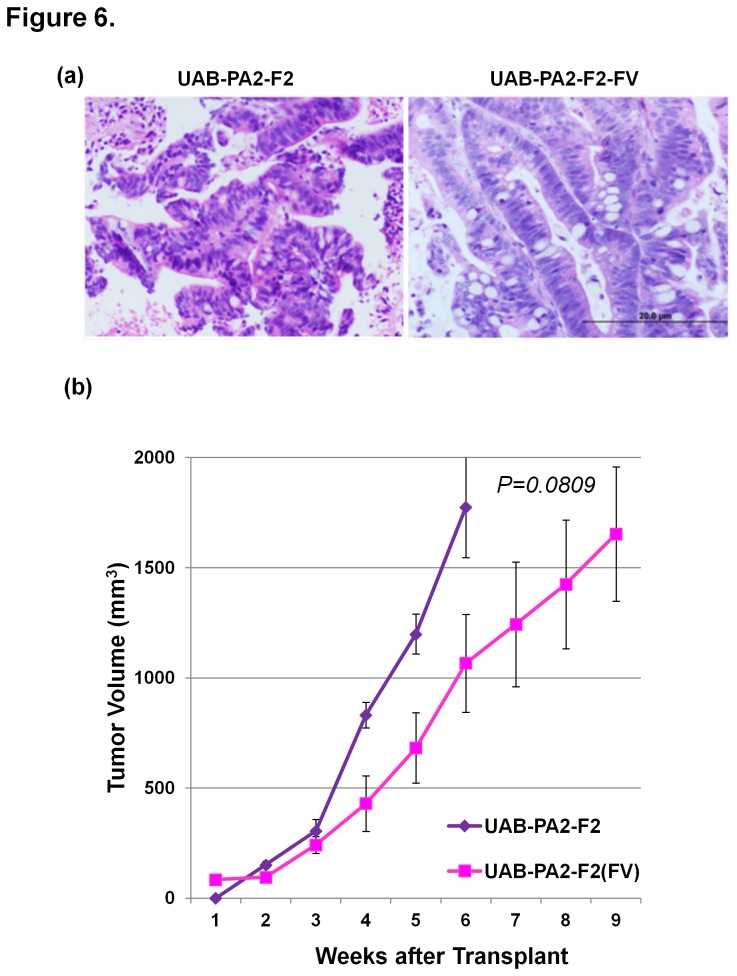
Cryopreservation of tumorgrafts does not alter the histology or growth kinetics in mice. (**a**) Photomicrographs of H&E stained sections of tumorgrafts produced from implantation of a primary tumor specimen within one hour of resection (UAB-PA2-F2)  compared to implantation of a specimen from the same tumor cryopreserved in liquid nitrogen for >30 days (UAB-PA2-F2-FV) show no histological differences. (**b**) Cryopreserved and fresh F0 tumor specimens produced tumorgrafts with similar growth kinetics in mice.  The apparent lag time of ~2 weeks prior to exponential growth of UAB-PA2-F2-FV tumorgrafts was not statistically significant (P = 0.0809).

Because it will be important to ensure the availability of tumorgraft models as novel chemotherapeutic agents become available for testing, we also examined whether cryopreserved F1 specimens formed tumors as readily as F1 specimens that had not been frozen. We compared *in vivo* growth characteristics of F2 tumors derived from frozen F1 specimens (UAB-PA2-F2-FV tumors) compared to fresh F1 specimens with the UAB-PA2 model. As outlined in Methods, after dissection, F1 specimens were either transplanted directly into mice (to produce UAB-PA2-F2) or stored in liquid nitrogen for a minimum of one month and then transplanted into mice to produce UAB-PA2-F2-FV tumors. H&E-stained sections of F2 tumors produced by both fresh and frozen F1 specimens showed the same degree of moderate differentiation and similar morphology: complex gland formation and increased N:C ratio with relatively intact nuclear polarity (Figure **6a**). The single difference noted between the two F2 generation progeny was that tumorgrafts from cryopreserved specimens had an apparent lag time of ~2 weeks (P=0.0809; Figure **6b**) compared to no apparent lag time for tumors derived from fresh specimens. 

### Primary tumor specimens that failed to produce tumors in mice showed distinct morphologies

**Figure 7:**
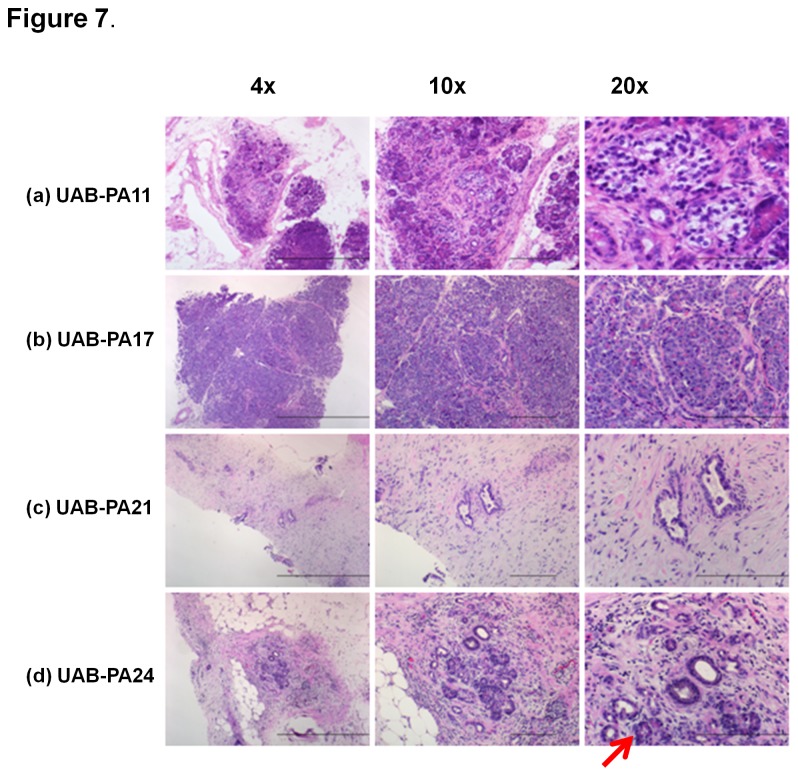
Histological evaluation of primary tumors that failed to grow in mice. Four primary tumor specimens (F0) that failed to produce F1 tumorgrafts were analyzed for their histological features using H&E staining. The photomicrographs show 4x, 10x, and 20x images of each tumorgraft. Bar in 4x = 100 µm; 10x = 20 µm; 20x = 20 µm.

Our primary tumor take rate in mice for pancreatic adenocarcinoma is 85%, higher than the previously reported 22-62% [38-40]. However, six of the original 41- F0 tumor specimens failed to produce tumors in mice. To determine whether these specimens represented a unique subset of PC tumors, we evaluated four of these six tumors for which we had sufficient FFPE tissue for analysis (Figure **7**). H&E stained sections of the first specimen (UAB-PA11) (Figure **7a**) shows F0 tissue containing benign pancreatic tissue with chronic pancreatitis, but no identifiable tumor cells (0% tumor content). The second specimen (UAB-PA17) (Figure **7b**), shows F0 tissue containing benign acinar pancreatic tissue, but no identifiable tumor cells (0% tumor content). The third specimen (UAB-PA21) (Figure **7c**) appears to contain some tumor cells (~10% of total cellular content), but is comprised primarily of stromal tissue. The fourth specimen (UAB-PA24) (Figure **7d**) shows focal suspicious glands in a degenerating pancreatic lobule against a background characteristic of chronic pancreatitis and remnant acinar tissue (red arrow), but no identifiable tumor cells (<1 % tumor content). These observations are consistent with the hypothesis that a critical ratio of tumor cells: stroma or a critical number of tumor cells is required to support growth in immunocompromised mice. In addition, the morphological features of specimens that differ from those successfully propagated in mice include fewer atypical glands and the presence of acini/stroma consistent with chronic inflammation. We concluded that the F0 specimens that did not produce tumors in mice had common histological features and a minimal percentage of malignant cells (0-10%). Of note, all primary specimens obtained from tumors of patients diagnosed with PDAC. Specimens that did not produce tumors when implanted into mice simply did not contain a sufficient number of tumor cells to produce tumors, emphasizing the heterogeneity of these specimens and the importance of verification of tumor histology prior to implant.  

## Discussion

The main goal of this study was to generate directly from patient tumor tissue, a panel of pancreatic ductal adenocarcinoma tumorgraft models that represent different disease stages and that reflect the heterogeneous nature of patient population. The main findings we report here are (i) histological fidelity in primary (F0), F1 and F2 specimens, based on H&E staining; (ii) genetic fidelity through at least two passages in immunocompromised mice, based on KRAS codon 12 mutation status; (iii) the observation that specimens derived from primary tumors at least 2.5 cm in greatest dimension have a higher take rate than smaller tumors; (iv) a higher tumor take rate in immunocompromised mice than has been reported previously [38-40]; and (v) histological data suggesting that successful engraftment may require that implants contain more than 10% tumor cells. 

Although a potential association between tumorgraft development and time between surgical resection and implantation is somewhat controversial [38], our data document that immediate (less than an hour post resection) implantation of F0 specimens into mice produced an apparent take rate of 85% (35 of 41 primary specimens successfully propagated in mice). We also demonstrated that four of the six specimens that failed to produce tumors in mice contained 0-10% tumor cells. Together these results indicate that the take rate for specimens containing >10% tumor cells approaches 95% (35/37). (Insufficient F0 material was available to analyze the remaining two specimens that failed to produce tumors in mice.) In addition to the impact of time to implantation and documentable presence of viable tumor cells on tumorgraft development, is the intriguing possibility that the presence of KRAS mutations influences the take rate of pancreatic tumors in preclinical models. Quite possibly, KRAS mutations confer a tumor cell survival advantage following resection. It may be difficult to address this hypothesis, however, since approximately 90% of resected pancreatic tumors harbor KRAS mutations [34,35]. 

Taken together, the comparison of our data with that in previous studies suggests that the major differences in methodology for our study was the minimal time between sample acquisition and implantation, and the gross examination by a pathologist of tumor specimens prior to implantation to evaluate the likelihood that viable tumor cells were present in the tissue. Our study also differed from several published studies in that primary tumor cells were not cultured *in vitro* prior to implantation into the mice, and no Matrigel or collagenase was used. These factors, in addition to rapid implantation, may also have affected take rate. Notably, our take rate of a minimum of 85% allows for evaluation of all stages of PDAC tumors, without introducing biases that might result if specimens from only particular tumor subclasses could be successfully propagated. Our models encompass all stages of PDAC (Table **1**).

We do note, however, while our models offer some advantages over others currently available, experimental design may contribute significantly to obtaining interpretable data. For example, in Figures **1**, **2**, and **S1** electropherogram tracings depicting results for F0 specimens were generated using human-specific PCR primers. Several of these tracings have attenuated, but readily discernible, peaks corresponding to nucleotide substitutions in the sequence of KRAS codon 12 of human DNA. This type of result is expected for DNA obtained from heterogeneous tissue, as demonstrated by Ogino, et al [31]. Further, excised surgical specimens likely contain normal and malignant human tissue, and tumorgraft specimens likely contain murine tissue as well. The use of PCR primers that differentiate between murine and human KRAS DNA sequences minimized the likelihood that contaminating murine DNA would obscure PCR results. Further, we consider it likely that successive generations of tumorgrafts will contain an increased amount of tumorgraft stroma of murine origin. Ongoing studies in our laboratory focused on identifying which tumorgraft generations that retain stromal components predominantly of human origin, an event that may affect tumorgraft growth and/or phenotype, are also being conducted with species-specific primers and antibodies essential to obtaining interpretable data. This consideration would apply to any of the available dual-species models. 

Methods used to establish patient-derived *in vivo* models differ somewhat among laboratories, but several have been used successfully to study specific aspects of PC. These studies fall into three categories. The first category is evaluation of the efficacy of existing or novel chemotherapeutic agents [41-43]. The second is identification of correlations between genotype or molecular phenotype and implantation take rate, treatment regimens, or patient survival [40]. And the third is exploration of the possibility of using this type of model to design personalized therapies [44,45]. These studies include, for example, extensive allelotyping to identify chromosomal loci that harbor tumor suppressor genes [46], or determination of the mutational status and expression level of SMAD4 specifically by tumor-generating PC cells [40]. The latter study concluded that PDAC tumor specimens with low level expression of SMAD4 protein loss had a statistically higher engraftment rate in mice (67%) than tumor specimens with normal SMAD4 protein levels (36%). Interestingly, these investigators also concluded that patients whose tumor specimens engrafted had a shorter duration of survival (299 days), than patients whose tumor specimens failed to grow in mice (>800 days), and that gemcitabine-resistant tumors displayed relatively high levels of cell adhesion molecules, focal adhesions, GAP junctions, and Notch signaling-associated proteins [40]. A third particularly noteworthy study, by Hidalgo et al, used tumorgraft models derived from 14 patients with refractory advanced pancreatic cancer to determine the efficacy of a myriad of treatment regimens in each model, and then to evaluate the utility of each model in identifying effective personalized treatment regimens for the patient from whom each model was derived [45]. Other laboratories have used similar models to identify c-Met as a marker for PC stem cells [47], to evaluate the drug sensitivity of PC stem cells [48], or to use gene signatures to predict the response of PC patient-derived *in vivo* models to specific cytotoxic agents [49]. These published studies show the utility of individual models to address specific questions regarding pancreatic cancer. However, no comprehensive histopathological analysis has been published, to verify the duration of genotypic or phenotypic fidelity to the tumor of origin. 

In summary, murine models of human solid tumors have emerged as particularly effective models to verify the causes of and evaluate treatments for human cancers. Among the types of models available, we propose that primary human tumorgrafts established directly from human tumors most accurately reflect morphologic, genetic and molecular characteristics of specific human tumors. The PDAC models we describe in this report reflect a cross section of all stages of pancreatic tumors considered to be resectable. The development of tumorgraft models documented to recapitulate the biology of human pancreatic adenocarcinoma will serve as valuable and necessary tools to identify and assess pathways and/or biomarkers critical to tumor progression and to develop effective therapies for this chemorefractory disease. 

## Supporting Information

Figure S1 **Mutations in codon 12 of the KRAS gene of primary PDAC tumors (F0) were conserved in the F1 and F2 tumorgrafts derived from each tumor.** Electropherograms show mutations in codon 12 of the KRAS gene in six primary PDAC tumors (F0) and in the F1 and F2 tumorgrafts derived from each tumor. Results for additional tumors are shown in Figure 1(c) and Table 3 of the main manuscript. Data for a total of 20 F0 tumors and corresponding F1 and F2 tumorgrafts are reported in this study.
(TIF)

Figure S2 **Histological evaluation of twelve F1 and F2 tumorgrafts demonstrates morphological fidelity of these tumors with the F0 tumors from which they were derived.** Histologic analyses are provided for a total of 20 F0 tumors and their corresponding F1 and F2 tumorgrafts in this study. See also Figure 3. **Tumor UAB-PA8 (j)**: Morphologic features are highly conserved from F0 through F2. The tumor remains moderately differentiated across generations with comparable gland formation in F0 and F1. F2 tumors display decreased, but recognizable, gland formation. Cytologically, F0 and F1 tumors display similar N:C ratios, with F2 showing decreased N:C ratio. There is a decreasing amount of peritumoral stroma across generations with F0 displaying the most and F2 the least. F0 through F2 are classified as PDAC. **Tumor UAB-PA13 (k)**: Tumor features are well conserved across generations (F0-F2). Morphologically, F0 through F2 tissues show moderate differentiation. The N:C ratio is preserved in F0 and F1, but decreased in F2. Also, the nuclear features of F2 are more dysplastic than those of F0 or F1. Peritumoral stroma appears decreased from F0 to F2. Interestingly, F1 has increased peritumoral adipose tissue compared to F0 and F2. F0 through F2 are classified as PDAC with PanIN-3 features. **Tumor UAB-PA20 (l)**: Tumor morphology appears conserved across generations in terms of maintained gland formation and similar amounts of peritumoral stroma. Cytologically, the tumor cell nuclei remain hyperchromatic and round across generations. However, the N:C ratio decreases significantly from F0 to F1, with a slight increase in F2. This may be secondary to reactive changes in F1 (mucin depletion). F1 displays features of PanIN-3/PDAC, while F1 and F2 display features of PDAC. **Tumor UAB-PA22 (m)**: Morphologically, the tumor remains moderately differentiated across generations. There is a progressive decrease in peritumoral stroma from F0 through F2. Cytologically, F1 appears to have decreased N:C ratio compared to F0 and F2. However, F1 appears to have more reactive epithelial changes (mucin depletion) which may explain the higher cytologic atypia. F2 displays increased N:C ratio with significant loss of nuclear polarity as well as decreased, though intact, gland formation. All generations display features of PDAC. **Tumor UAB-PA26 (n)**: The primary tumor appears moderately to poorly differentiated while F1 and F2 generations appear moderately differentiated. The amount of peritumoral stroma appears to vary from F0 to F2. Cytologically, all exhibit very increased N:C ratio compared to cytology expected for normal glandular mucosa with moderate nuclear atypia. All exhibit features of PDAC. **Tumor UAB-PA28 (o)**: The tumor appears moderately differentiated across all generations. There appears to be a progressive decrease in peritumoral stroma from F0 to F2. Cytologically, the tumor seems to have maintained a high N:C ratio across all generations with conserved atypical nuclear features (hyperchromatic, loss of polarity). All generations display features of PDAC. **Tumor UAB-PA29 (p)**: Interestingly, this tumor appears to have progressed from moderately to poorly differentiated in F0 to moderately differentiated in F1 and F2. There appears to be a decreasing amount of peritumoral stroma from F0 to F2. Cytologically, F1 and F2 tumor cells appear to have a lower N:C ratio than F0. Both F1 and F2 tumors have better gland formation than F0, and F2 has improved gland formation over F1. All have features of PDAC, with F1 showing PDAC/PanIN-3 features. **Tumor UAB-PA30 (q)**: The tumor has similar characteristics from F0 to F2 and remains moderately differentiated across generations. There appears to be more peritumoral stroma in F0 and F1 than in F2. Cytologically, the degree of nuclear atypia appears stable from F0 to F2, while the N:C ratio appears to decrease slightly from one generation to the next. All display characteristics of PDAC. **Tumor UAB-PA33 (r)**: The tumor appears well differentiated in F0 and moderately differentiated in F1 and F2. There appears to be a significant decrease in peritumoral stroma from F0 to F2. Cytologically both nuclear and atypia and N:C ratio increases from F0 to F2. All display characteristics of PDAC. **Tumor UAB-PA34 (s)**: F0 has more peritumoral stroma and smaller tumor load than either F1 and F2. Though there are fewer tumor cells/glands, the F0 tumor displays characteristics of moderate differentiation, similar to F1 and F2 tumors. There is a decrease in peritumoral stroma from F1 to F2. Cytologically, F2 tumors appear to have increased nuclear atypia compared to F0 and F1. The N:C ratio appears variable across generations, with F1 having lower N:C ratio than F0 and F2. All display characteristics of PDAC. **Tumor UAB-PA36 (t)**: The F0 generation tumor is moderately to poorly differentiated, while F1 and F2 tumors are moderately differentiated. F1 and F2 tumors have more peritumoral stroma than F0, although there seems to be a decrease between F1 and F2. The cytology of F0 is more high grade than F1 and F2 in terms of nuclear atypia and N:C ratio. All display characteristics of PDAC, with F1 displaying features of PDAC/PanIN-3. **Tumor UAB-PA37 (u)**: The F0 generation appears moderately to poorly differentiated compared to F1 and F2, both of which appear moderately differentiated. There is slightly more peritumoral stroma in F1 and F2 than in F0. In terms of cytology, both F1 and F2 have lower grade nuclear atypia and N:C ratio than F0. All display characteristics of PDAC.
(TIF)

Figure S3 **(a). Growth curves for six first generation (F1) tumorgrafts.** See Figures 5(a) and S3(c) for growth curves of additional F1 tumorgrafts. **(b). Growth curves for six second generation (F2) tumorgrafts.** See Figures 5(b) and S3(d) for growth curves of additional F2 tumorgrafts. **(c). Growth curves for six first generation (F1) tumorgrafts.** See Figures 5(a) and S3(a) for growth curves of additional F1 tumorgrafts. **(d). Growth curves for six second generation (F2) tumorgrafts.** See Figures 5(b) and S3(b) for growth curves of additional F2 tumorgrafts.
(TIF)
