# Supplementary figures and images for: Development and Histopathological Characterization of Tumorgraft Models of Pancreatic Ductal Adenocarcinoma
Source: PLoS One. 2013 Oct 23;8(10):e78183. doi: 10.1371/journal.pone.0078183 (PMC3806809; doi:10.1371/journal.pone.0078183)

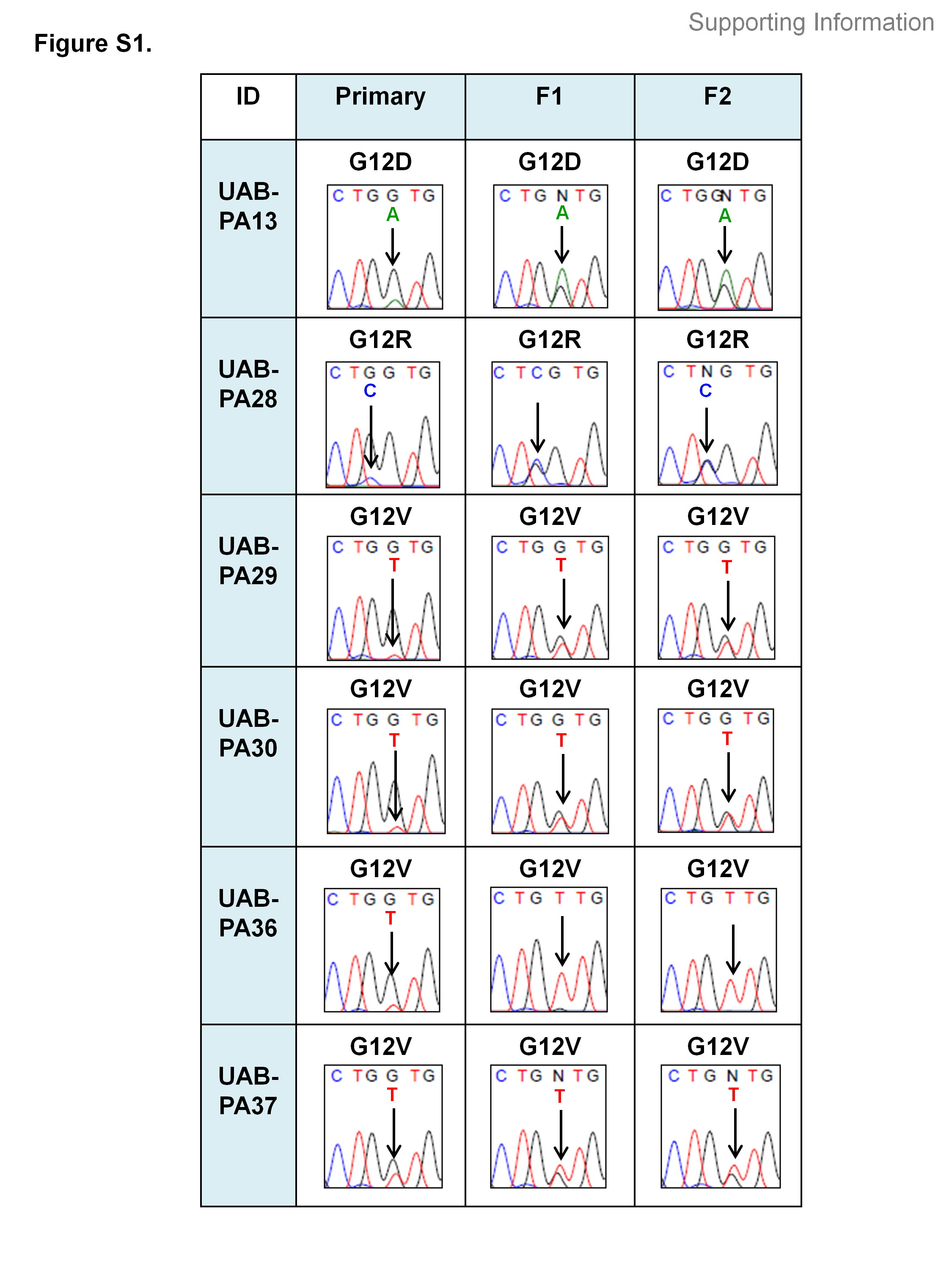

Supplement: Figure S1 — Mutations in codon 12 of the KRAS gene of primary PDAC tumors (F0) were conserved in the F1 and F2 tumorgrafts derived from each tumor. Electropherograms show mutations in codon 12 of the KRAS gene in six primary PDAC tumors (F0) and in the F1 and F2 tumorgrafts derived from each tumor. Results for additional tumors are shown in Figure 1(c) and Table 3 of the main manuscript. Data for a total of 20 F0 tumors and corresponding F1 and F2 tumorgrafts are reported in this study. (TIF) [file pone.0078183.s001.tif]

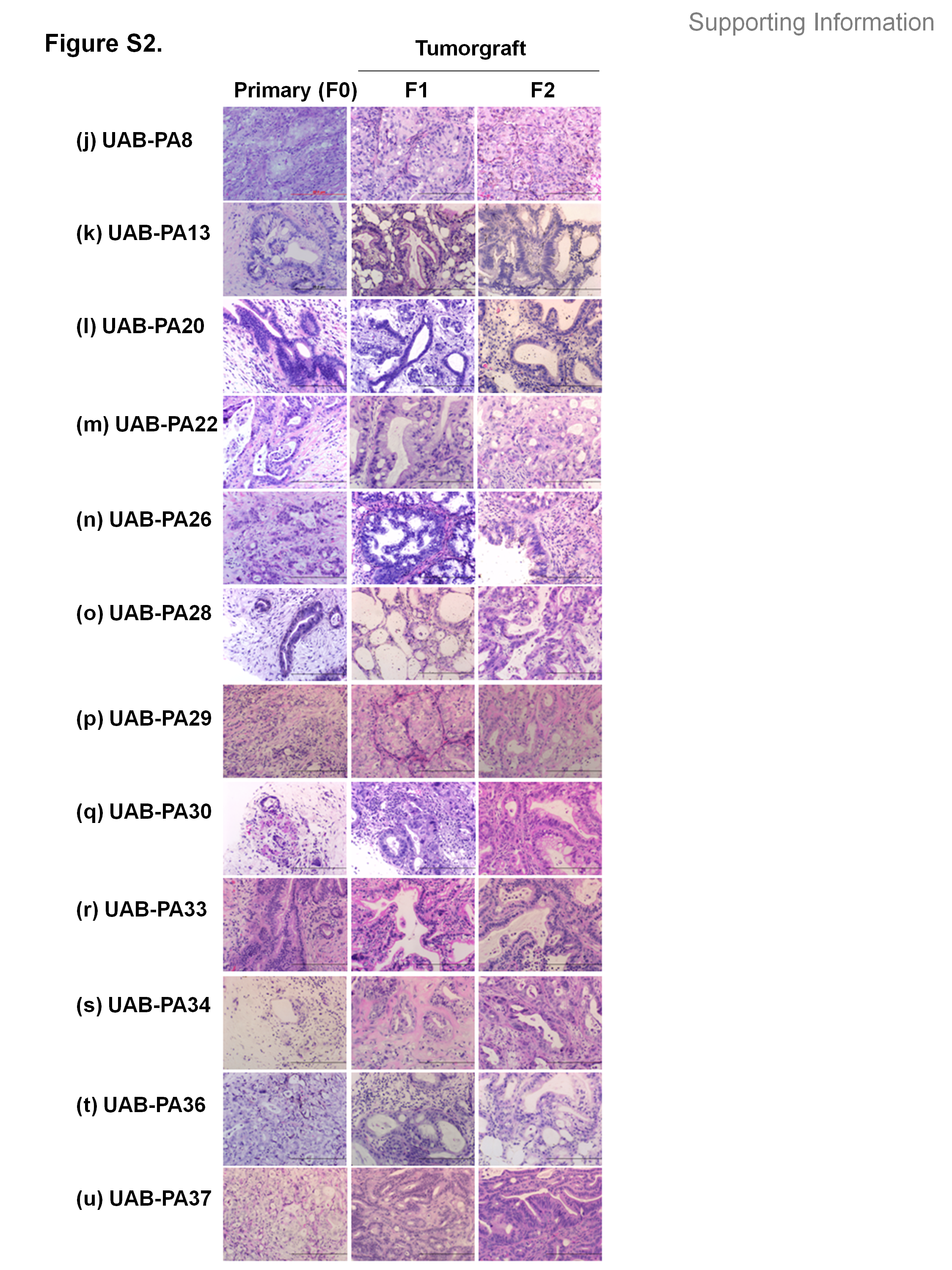

Supplement: Figure S2 — Histological evaluation of twelve F1 and F2 tumorgrafts demonstrates morphological fidelity of these tumors with the F0 tumors from which they were derived. Histologic analyses are provided for a total of 20 F0 tumors and their corresponding F1 and F2 tumorgrafts in this study. See also Figure 3. Tumor UAB-PA8 (j): Morphologic features are highly conserved from F0 through F2. The tumor remains moderately differentiated across generations with comparable gland formation in F0 and F1. F2 tumors display decreased, but recognizable, gland formation. Cytologically, F0 and F1 tumors display similar N:C ratios, with F2 showing decreased N:C ratio. There is a decreasing amount of peritumoral stroma across generations with F0 displaying the most and F2 the least. F0 through F2 are classified as PDAC. Tumor UAB-PA13 (k): Tumor features are well conserved across generations (F0-F2). Morphologically, F0 through F2 tissues show moderate differentiation. The N:C ratio is preserved in F0 and F1, but decreased in F2. Also, the nuclear features of F2 are more dysplastic than those of F0 or F1. Peritumoral stroma appears decreased from F0 to F2. Interestingly, F1 has increased peritumoral adipose tissue compared to F0 and F2. F0 through F2 are classified as PDAC with PanIN-3 features. Tumor UAB-PA20 (l): Tumor morphology appears conserved across generations in terms of maintained gland formation and similar amounts of peritumoral stroma. Cytologically, the tumor cell nuclei remain hyperchromatic and round across generations. However, the N:C ratio decreases significantly from F0 to F1, with a slight increase in F2. This may be secondary to reactive changes in F1 (mucin depletion). F1 displays features of PanIN-3/PDAC, while F1 and F2 display features of PDAC. Tumor UAB-PA22 (m): Morphologically, the tumor remains moderately differentiated across generations. There is a progressive decrease in peritumoral stroma from F0 through F2. Cytologically, F1 appears to have decreased N:C ratio [file pone.0078183.s002.tif]

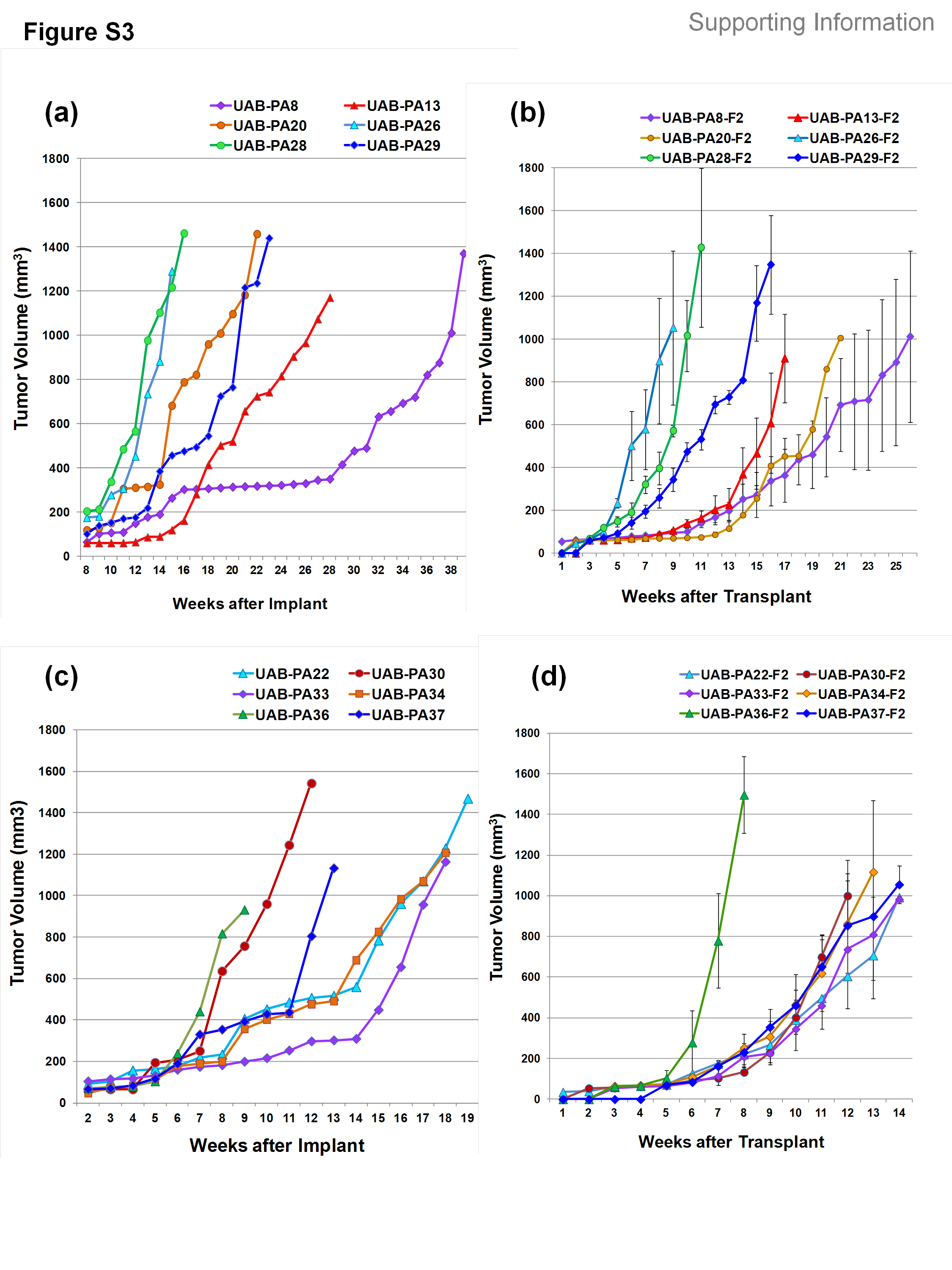

Supplement: Figure S3 — (a). Growth curves for six first generation (F1) tumorgrafts. See Figures 5(a) and S3(c) for growth curves of additional F1 tumorgrafts. (b). Growth curves for six second generation (F2) tumorgrafts. See Figures 5(b) and S3(d) for growth curves of additional F2 tumorgrafts. (c). Growth curves for six first generation (F1) tumorgrafts. See Figures 5(a) and S3(a) for growth curves of additional F1 tumorgrafts. (d). Growth curves for six second generation (F2) tumorgrafts. See Figures 5(b) and S3(b) for growth curves of additional F2 tumorgrafts. (TIF) [file pone.0078183.s003.tif]
